# Long‐term quality of life and cost‐effectiveness of treatment of partial thickness burns: A randomized controlled trial comparing enzyme alginogel vs silver sulfadiazine (FLAM study)

**DOI:** 10.1111/wrr.12799

**Published:** 2020-02-11

**Authors:** Zjir M. Rashaan, Pieta Krijnen, Kelly AA Kwa, Margriet E. van Baar, Roelf S. Breederveld, M. Elske van den Akker‐van Marle

**Affiliations:** ^1^ Department of Surgery Leiden University Medical Centre Leiden Netherlands; ^2^ Department of Surgery Red Cross Hospital Beverwijk Netherlands; ^3^ Burn Centre, Red Cross Hospital Beverwijk Netherlands; ^4^ Association of Dutch Burn Centres, Maasstad Hospital Rotterdam Netherlands; ^5^ Department of Public Health, Erasmus MC University Medical Center Rotterdam Rotterdam Netherlands; ^6^ Department of Biomedical Data Sciences, Medical Decision Making Leiden University Medical Centre Leiden Netherlands

## Abstract

The clinical effectiveness and scar quality of the randomized controlled trial comparing enzyme alginogel with silver sulfadiazine (SSD) for treatment of partial thickness burns were previously reported. Enzyme alginogel did not lead to faster wound healing (primary outcome) or less scar formation. In the current study, the health‐related quality of life (HRQoL), costs, and cost‐effectiveness of enzyme alginogel compared with SSD in the treatment of partial thickness burns were studied. HRQoL was evaluated using the Burn Specific Health Scale‐Brief (BSHS‐B) and the EQ‐5D‐5L questionnaire 1 week before discharge and at 3, 6, and 12 months postburn. Costs were studied from a societal perspective (health care and nonhealth‐care costs) for a follow‐up period of 1 year. A cost‐effectiveness analysis was performed using cost‐effectiveness acceptability curves and comparing differences in societal costs and Quality Adjusted Life Years (QALYs) at 1 year postburn. Forty‐one patients were analyzed in the enzyme alginogel group and 48 patients in the SSD group. None of the domains of BSHS‐B showed a statistically significant difference between the treatment groups. Also, no statistically significant difference in QALYs was found between enzyme alginogel and SSD (difference −0.03; 95% confidence interval [CI], −0.09 to 0.03; *P* = .30). From both the health care and the societal perspective, the difference in costs between enzyme alginogel and SSD was not statistically significant: the difference in health‐care costs was €3210 (95% CI, €‐1247 to €7667; *P* = .47) and in societal costs was €3377 (95% CI €‐6229 to €12 982; *P* = .49). The nonsignificant differences in costs and quality‐adjusted life‐years in favor of SSD resulted in a low probability (<25%) that enzyme alginogel is cost‐effective compared to SSD. In conclusion, there were no significant differences in quality of life between both treatment groups. Enzyme alginogel is unlikely to be cost‐effective compared with SSD in the treatment of partial thickness burns.

## 1 INTRODUCTION

The optimal treatment of partial thickness burns remains an unsolved challenge in the absence of a gold standard treatment.^1^, ^2^, ^3^ The available literature is mainly based on clinical studies of poor quality that report mostly on clinical outcomes (eg, wound healing) and incidentally on scar quality.^1^, ^4^, ^5^ Therefore, there is a need for well‐designed trials that not only evaluate clinical outcomes and scar formation but also health‐related quality of life (HRQoL), costs, and cost‐effectiveness to help establish optimal treatment of partial thickness burns.

Two retrospective studies showed faster wound healing when enzyme alginogel, which is a hydrated alginates polymers in a polyethyleneglycol (PEG) matrix embedded with a biologic enzyme system of glucose oxidase, lactoperoxidase and guaiacol was compared with SSD in the treatment of partial thickness burns, while no data were available with regard to scar formation, HRQoL, costs, or cost‐effectiveness.^6^, ^7^ Therefore, our research group performed a randomized controlled trial (RCT) comparing enzyme alginogel with SSD in the treatment of partial thickness burns (FLAM study).^8^ Enzyme alginogel was not found to be superior with regard to clinical outcomes such as wound healing time (primary outcome), pain, incidence of infection, and scar quality, although patients in the enzyme alginogel group required significantly less dressing changes compared with the SSD group.^9^ Less dressing changes in the enzyme alginogel group were expected to lead to less treatment costs compared with the SSD group. In this light, HRQoL, costs, and cost‐effectiveness of the treatment modalities might be decisive factors for choosing between the two treatments in clinical practice. Therefore, this study evaluated the HRQoL, costs, and cost‐effectiveness of enzyme alginogel compared with SSD in the treatment of partial thickness burns.

## 2 MATERIAL AND METHODS

### 2.1 Study design

Patients with partial thickness burns participated in an open label, multicenter RCT comparing the clinical effectiveness, quality of life, and costs of enzyme alginogel with SSD. The detailed study protocol was published previously.^8^ The study was approved by the Medical Research Ethics Committee Noord‐Holland (NL43671.094.13) and conducted at two Dutch Burn Centers (Red Cross Hospital, Beverwijk and Maasstad Hospital, Rotterdam) from February 2014 until September 2015. Patients were eligible for the study if they were 18 years or older; had partial thickness burns of minimally 1% affected total body surface area (TBSA); presented within 48 hours of the burn injury; were mentally competent or temporary incompetent (because of sedation and/or intubation); and provided written informed consent. Patients were excluded if they had TBSA >30%; burns caused by chemicals, electricity, or radiation; if local therapy had already started; or if the treating physician expected the patients not to be compliant with the study protocol. The patients were randomly allocated to treatment with either Flaminal Forte (Flen Pharma, Belgium), which is an enzyme alginogel consists of 5.5% hydrated alginates and a biologic antimicrobial system (glucose oxidase, lactoperoxidase, and guaiacol) or Flamazine (Sinclair Pharmaceuticals, Surrey, United Kingdom) which consists of silver sulfadiazine (SSD) 10 mg/g in hydrophilic crème base.

### 2.2 Time to wound healing and operation

In addition to previously published results on clinical effectiveness of the treatment modalities in the FLAM study,^9^ of the results for time to wound healing and need for operation were analyzed in subgroups of patients with different wound depths, based on results of the Laser Doppler imager in combination with the clinical diagnosis.^10^, ^11^ From a clinical point of view, stratification of different wound depths of partial thickness wounds is important because superficial and intermediate partial thickness burns are likely to heal spontaneously in less than 3 weeks, while deep partial thickness burns often require operation.^11^

### 2.3 Health‐related quality of life

HRQoL was evaluated using the Dutch version of the Burn Specific Health Scale‐Brief (BSHS‐B) and the EQ‐5D‐5L questionnaire 1 week before discharge and at 3, 6, and 12 months postburn. The BSHS‐B is a valid and reliable self‐administered questionnaire with 40 items that cover nine domains: simple abilities, heat sensitivity, hand function, treatment regimens, work, body image, affect, interpersonal relationships and sexuality. All items are scored on a scale from 0 (extreme difficulty) to 4 (no difficulty at all).^12^, ^13^

The EQ‐5D‐5L is a generic quality of life questionnaire, which is widely used in economic evaluations, because it enables the comparison of quality of life outcomes for all kinds of interventions and different diseases. The questionnaire comprises two components.^14^ The first is a descriptive system that defines health states based on five dimensions: mobility, self‐care, usual activities, pain/discomfort, and anxiety/depression. Each dimension is scored with one item on five levels ranging from no problems to extreme problems. The combination of the scores for the five dimensions can be translated to utility values, ranging from 0 (health as bad as death) to 1 (perfect health), based on a so‐called tariff, which is obtained by the valuation of the Dutch population for the different health states.^15^ The second component is a Visual Analogue Scale (VAS), on which the burn patients rate their health state, ranging from 0 (worst imaginable health state) to 100 (perfect health). The VAS score can also be transformed to a utility value using the power transformation 1‐(1‐VAS/100)^1.61^.^16^

Quality adjusted life years (QALYs) were used to evaluate the cost‐effectiveness over a period of 12 months. QALYs combine EQ‐5D‐5L and EQ‐VAS utilities values with duration of the follow‐up period.^17^ QALYs were calculated from the area‐under‐the‐curve method of the utilities obtained from the EQ‐5D during the 12 months of follow‐up.^14^

### 2.4 Costs

Costs were studied from the societal perspective, which included both health‐care costs in and outside the hospital and nonhealth‐care costs (productivity loss and travel costs). Data on health‐care use were recorded prospectively by the FLAM study research team as part of the case record form during admission and by means of patient questionnaires at 3, 6, and 12 months postburn. Costs were calculated by multiplying the volumes of health‐care use by the corresponding unit prices. Because of the 1‐year time horizon, costs were not discounted. Costs were expressed in Euros and converted to the 2018 price level using the general Dutch consumer price index.^18^

#### 2.4.1 *Treatment*

Costs of treatment were determined by microcosting, taking into account used materials and personnel time. To assess costs of wound care, material and personnel time (ICU and non‐ICU nurse) needed for each dressing change were recorded daily for each patient. The unit price for materials was obtained from the financial department of the Red Cross Hospital, Beverwijk. Subsequently, total material costs were calculated for each patient. Personnel time needed for each dressing change was recorded in hours. Costs of personnel time per hour were based on the gross salary of the nurses, increased with a surcharge for holiday allowance and social charges.^19^ Personnel, material, and equipment costs of surgery were obtained from a previous Dutch study by Hop et al.^20^ Personnel costs were multiplied by time (surgical and anesthesia team) needed for each operation recorded in the current study. For each patient, information on reconstructive surgery, use of blood products, pressure clothes and silicone therapy were recorded prospectively during hospital admission and the follow‐up period up to 12 months postburn. The unit price for the reconstructive surgery was derived from a previous Dutch study on this subject.^21^ Unit prices of blood products, pressure clothes, and silicone therapy were derived from the financial department and supplier.

#### 2.4.2 *Diagnostics and clinical consultations during hospitalization*

Diagnostic procedures included bronchoscopy, swabs, laboratory tests, and radiology, which were recorded daily during admission. Unit prices of these diagnostic procedures were obtained from the Dutch manual for costing in economic evaluation and the Dutch Healthcare Authority.^19^, ^22^

#### 2.4.3 *Burn center stay and outpatient burn care*

Length of burn center stay in days and number of outpatient burn care visits during the follow‐up period of 12 months postburn were recorded on the case record forms. Burn center stay in days included days spent in the Intensive care Unit (ICU) of the burn center, non‐ICU burn center days and readmittance days. Unit costs were obtained from a previous Dutch study by Hop et al.^23^ Other health‐care use (rehabilitation, nursing home, visits to general practitioners, and allied health‐care professionals outside the hospital) was assessed by questionnaires during follow‐up period of 12 months. Unit costs were obtained from the Dutch manual for costing in economic evaluation.^19^

#### 2.4.4 *Nonhealth‐care costs*

Nonhealth‐care costs included costs of loss of economic productivity due to absence from work (by both patients and partner) and travel costs. Data on work absence were collected by questionnaires from the patients at 3, 6, and 12 months postburn. Productivity losses were valued using the friction cost method.^24^

## 3 STATISTICAL ANALYSIS

All analyses followed the intention‐to‐treat principle. All statistical analyses were conducted with IBM SPSS Statistics for Windows, version 22 (IBM Corp., Armonk, N.Y., USA). BSHS results were presented as median, while utility values and costs were presented as mean. Furthermore, a two‐sided *t*‐test or Mann‐Whitney test was used for comparing continuous data, and a two‐sided Chi‐square test or Fisher's exac*t* test for categorical data.

For the cost‐effectiveness analysis, multiple imputation by chained equations was used to reduce possible bias caused by missing data. Missing utility values or cost items were imputed using a switching regression model that included age, gender, TBSA, location of the study area and randomization group. Cost and QALYs were compared using the net benefit approach.^25^ Depending on the willingness to pay for a QALY, a strategy is cost‐effective compared with an alternative strategy if it has a higher net benefit (willingness to pay × QALYs ‐ costs). Cost‐effectiveness acceptability curves depict the probability that a strategy is cost‐effective as a function of willingness to pay, given the statistical uncertainty in costs and QALYs. The threshold of willingness to pay that is commonly accepted in the Netherlands is between €20 000 and € 80 000 per QALY, depending on disease burden.^26^ The base‐case cost‐utility analysis compared QALYs at 1 year on the basis of the EQ‐5D‐5L (Dutch tariff). Sensitivity analyses were carried out using the EQ‐VAS as a utility measure.

## 4 RESULTS

### 4.1 Study population

Of the 90 included patients, 89 patients were analyzed. One patient in the enzyme alginogel group discontinued participation in the trial during the admission period. The treatment groups were comparable with regard to age, gender, percentage of TBSA of the study area, trauma mechanism and anatomical location of the study area (Table 1). Lost to follow‐up were 4/41 (10%) patients in the enzyme alginogel group and 3/48 (6%) patients in the SSD group.

**Table 1:** Characteristics of patients

Characteristic	Enzyme alginogel (n = 41)	Silver sulfadiazine (n = 48)
Age in years, mean (SD)	50 (15)	43 (16)
Male gender, n (%)	32 (78)	39 (81)
**%**TBSA study area, median (range)		
Partial thickness burns	3 (1‐10)	3 (1‐16)
Superficial and/ or intermediate	2 (1‐9)	2 (1‐9)
Deep^a^	2 (2‐10)	4 (1‐16)
Trauma mechanism, n (%)		
Scald	4 (10)	7 (15)
Flame	20 (49)	21 (44)
Flash	12 (29)	16 (33)
Hot grease	2 (5)	4 (8)
Steam	3 (7)	0 (0)
Location of study area, n (%)		
Head and neck	1 (2)	1 (2)
Trunk (anterior)	10 (24)	6 (13)
Trunk (posterior)	6 (15)	2 (4)
Upper extremities	16 (39)	24 (50)
Lower extremities	8 (20)	15 (31)

a Burn wounds with deep partial thickness burns as the deepest wound depth.

### 4.2 Time to wound healing and operation

As represented in Table 2, the median time to wound healing and need for operation did not differ between the enzyme alginogel group and the SSD group, neither within the subgroup of patients with superficial and/or intermediate partial thickness buns nor in the subgroup of patients with deep partial thickness burns.

**Table 2:** Time to wound healing and need for operation based on burn wound depth of the partial thickness burns

Outcome measure	Enzyme alginogel (n = 41)	Silver sulfadiazine (n = 48)	*P*
*Superficial and/or intermediate partial thickness burns*			
Time to wound healing (days), median (range), n	15 (8‐32) n = 19	12 (7‐27) n = 22	.08^a^
Need for operation, n (%)	5/19 (26%)	5/22 (23%)	.89^b^
*Deep partial thickness burns* c			
Time to wound healing (days), median (range), n	19 (11‐49) n = 22	18 (11‐48) n = 26	.92^a^
Need for operation, n (%)	16/22 (73%)	19/26 (73%)	.79^b^

a Mann‐Whitney test.

b Chi‐square test.

c Burn wounds with deep partial thickness burns as the deepest wound depth.

### 4.3 Quality of life

For all nine domains of the BSHS‐B, the amount of perceived problems decreased after hospital discharge. No statistically significant or clinically relevant differences between the treatment groups were found in any of the nine domains of BSHS‐B at any follow‐up moment (Table 3). The utility values for the patients' health states according to the Dutch EQ‐5D‐5L and EQ‐VAS at 3, 6, and 12 months also showed no statically significant or clinically relevant differences between the treatment groups (Table 4). The mean QALYs based on the EQ‐5D‐5L results over the 12 months postburn were 0.81 for enzyme alginogel group and 0.84 for SSD group. The difference in mean QALYs was not statistically significant (−0.03; 95% confidence interval [CI] −0.09 to 0.03; *P* = .30). The mean QALYs obtained using the VAS over the study period were 0.89 for enzyme alginogel group and 0.90 for SSD group. The difference in mean QALYs of EQ‐VAS was not statistically significant (−0.01; 95% CI: −0.05 to 0.02; *P* = .42).

**Table 3:** Scores on the Burn Specific Health Scale (BSHS)‐Brief during follow‐up of 12 months

	Enzyme alginogel			silver sulfadiazine			
	No.	Median	Range	No.	Median	Range	*P* a
Simple abilities							
During admission	38	2.7	0.0‐4.0	44	2.8	0.0‐4.0	.21
3 months postburn	35	4.0	0.3‐4.0	41	4.0	0.0‐4.0	.43
6 months postburn	34	4.0	0.0‐4.0	38	4.0	0.0‐4.0	.08
12 months postburn	34	4.0	0.0‐4.0	36	4.0	3.7‐4.0	.08
Heat sensitivity							
During admission	36	2.8	0.0‐4.0	35	3.0	0.4‐4.0	.32
3 months postburn	34	3.5	0.2‐4.0	42	3.4	0.0‐4.0	.77
6 months postburn	34	3.6	1.8‐4.0	39	3.8	0.8‐4.0	.14
12 months postburn	34	3.6	1.8‐4.0	36	3.8	1.4‐4.0	.40
Hand function							
During admission	38	3.2	0.0‐4.0	44	3.2	0.0‐4.0	.98
3 months postburn	35	4.0	1.0‐4.0	41	4.0	0.0‐4.0	.99
6 months postburn	34	4.0	0.0‐4.0	38	4.0	0.0‐4.0	.37
12 months postburn	34	4.0	0.0‐4.0	36	4.0	2.8‐4.0	.17
Treatment regimens							
During admission	37	3.2	0.2‐4.0	33	3.2	0.0‐4.0	.42
3 months postburn	34	3.8	0.2‐4.0	42	4.0	0.8‐4.0	.86
6 months postburn	34	4.0	2.0‐4.0	39	4.0	2.2‐4.0	.80
12 months postburn	34	4.0	2.0‐4.0	36	4.0	0.8‐4.0	.38
Work							
During admission	36	2.0	0.0‐4.0	40	1.1	0.0‐4.0	.28
3 months postburn	35	3.3	0.0‐4.0	42	3.1	0.0‐4.0	.71
6 months postburn	34	3.6	0.5‐4.0	39	3.8	0.0‐4.0	.47
12 months postburn	34	4.0	2.3‐4.0	34	4.0	0.0‐4.0	.18
Body image							
During admission	37	3.5	0.0‐4.0	42	3.0	0.5‐4.0	.34
3 months postburn	35	3.7	0.0‐4.0	42	3.7	1.3‐4.0	.69
6 months postburn	34	3.9	0.8‐4.0	39	3.8	0.8‐4.0	.61
12 months postburn	34	4.0	1.0‐4.0	36	3.9	0.3‐4.0	.63
Affect							
During admission	37	3.4	1.0‐4.0	43	3.6	1.1‐4.0	.99
3 months postburn	35	3.7	1.0‐4.0	42	4.0	1.4‐4.0	.28
6 months postburn	34	4.0	0.7‐4.0	39	4.0	2.7‐4.0	.34
12 months postburn	34	4.0	2.8‐4.0	36	4.0	2.4‐4.0	.08
Interpersonal relationships							
During admission	37	3.5	0.0‐4.0	40	4.0	1.0‐4.0	.09
3 months postburn	34	4.0	1.8‐4.0	41	4.0	1.0‐4.0	.66
6 months postburn	34	4.0	0.5‐4.0	39	4.0	2.8‐4.0	.56
12 months postburn	34	4.0	1.5‐4.0	35	4.0	3.5‐4.0	.42
Sexuality							
During admission	36	4.0	0.0‐4.0	38	4.0	1.3‐4.0	.96
3 months postburn	35	4.0	0.0‐4.0	42	4.0	0.0‐4.0	.91
6 months postburn	34	4.0	0.3‐4.0	39	4.0	2.0‐4.0	.26
12 months postburn	34	4.0	2.3‐4.0	35	4.0	2.3‐4.0	.51

a Mann‐Whitney test.

**Table 4:** Utility values after treatment with enzyme alginogel and Silver sulfadiazne. Results are expressed as mean (SE of the mean)

Measure	Enzyme alginogel (n = 41)	Silver sulfadiazine (n = 48)	Difference	*P* a
EQ‐5D‐5L Dutch, utilities				
During admission	0.57	0.53	0.04 (−0.08‐0.16)	.52
3 months postburn	0.80	0.84	−0.04 (−0.13‐0.04)	.30
6 months postburn	0.84	0.89	−0.05 (−0.12‐0.02)	.19
12 months postburn	0.89	0.92	−0.03 (−0.08‐0.03)	.30
EQ‐VAS, utilities				
During admission	0.75	0.78	−0.03 (−0.11‐0.05)	.46
3 months postburn	0.89	0.89	−0.001 (−0.05‐0.05)	.98
6 months postburn	0.91	0.92	−0.01 (−0.05‐0.03)	.56
12 months postburn	0.92	0.94	−0.02 (−0.05‐0.01)	.10

EQ‐5D‐5L Dutch, utilities: utilities obtained from EQ 5‐D‐5L (Dutch tariff); EQ‐VAS, utilities: utilities obtained from EQ Visual Analogue Scale using the power transformation 1‐(1‐VAS/100)^1.61^.

a *t* test.

### 4.4 Health‐care costs

The mean costs of treatment per patient, including wound care, operation and scar therapy, were €4352 for the enzyme alginogel group and €3712 for the SSD group (Table 5). The difference in mean costs was not statistically significant (€640; 95% CI: €‐769 to €2049; *P* = .37). The mean of total healthcare costs per patient, including treatment, diagnostic procedures, clinical consultations, burn center stay, outpatient burn care and other health‐care costs was €31 031 for the enzyme alginogel group and €27 821 for the SSD group, which were not statistically different (difference: €3210; 95% CI: €1247 to €7667; *P* = .47). Burn center stay costs represented the largest part of healthcare costs (63% in the enzyme alginogel group and 69% in the SSD group), followed by treatment costs (14% in the enzyme alginogel group 14% and 13% in the SSD group).

**Table 5:** Mean costs of health care and nonhealth‐care costs in € (2018) per patient

	Enzyme alginogel (n = 41)		Silver sulfadiazine (n = 48)		Difference	
	Proportion of patients	Costs	Proportion of patients	Costs	Costs (95% confidence interval)	*P*
*Treatment*						
Wound care	1.00	2481	1.00	2156	325 (−458 to 1108)	.42
Surgical treatment^a^	0.54	1638	0.52	1210	429 (−265 to 1123)	.23
Blood products (erythrocytes)	0.07	0.94	0.08	0.61	0.34 (−1 to 2)	.68
Pressure garments	0.41	211	0.52	329	−119 (−311 to 74)	.23
Silicon therapy	0.20	10	0.25	10	0.04 (−10 to 10)	.99
Splints	0.10	11	0.04	6	5 (−9 to 18)	.51
Total treatment	1.00	4352	1.00	3712	640 (−769 to 2049)	.37
*Diagnostic procedures*						
Swabs	0.98	585	1.00	565	20 (−152 to 192)	.82
Lab tests	0.66	77	0.75	92	−16 (−95 to 64)	.70
Bronchoscopy	0.07	61	0.04	17	44 (−33 to 120)	.27
Radiology	0.32	75	0.40	92	−17 (−105 to 71)	.71
Others	0.20	12	0.21	23	−10 (−30 to 10)	.31
Total diagnostic procedures	0.98	810	1.00	789	21 (−314 to 356)	.90
*Clinical consultations*						
Physiotherapist	0.78	40	0.90	45	−5 (−22 to 12)	.54
Occupational therapist	0.56	22	0.56	30	−8 (−23 to 7)	.31
Social worker	0.29	26	0.29	32	−7 (−34 to 22)	.63
Dietitian	0.27	9	0.38	11	−2 (−10 to 6)	.62
Psychologist	0.27	17	0.13	8	10 (−3 to 23)	.15
Skin therapist	0.00	0.00	0.02	0.21	−0.21 (−0.61 to 0.20)	.32
Psychiatrist	0.12	45	0.06	42	3 (−80 to 87)	.94
Speech therapist	0.07	4	0.02	2	2 (−3 to 7)	.44
Rehabilitation physician	0.02	5	0.04	3	0.55 (−8 to 9)	.90
*Total clinical consultations*	0.90	167	0.98	174 177	−10 (−119 to 99)	.85
*Burn center stay*						
Non‐ICU burn center days	1.00	15 044	1.00	14 737	307 (−3110 to 3724)	.86
ICU burn center days	0.12	4271	0.29	4112	159 (−4408 to 4725)	.95
Re‐admittance days	0.05	348	0.04	233	114 (−418 to 647)	.67
Day care	0.05	10	0.04	63	−52 (−108 to 3)	.35
Total burn center stay	1.00	19 672	1.00	19 145	527 (527 to 527)	1.00
*Outpatient burn care*						
Outpatient wound care	0.88	240	0.92	226	15 (−88 to 117)	.78
Outpatient scar care	0.95	328	0.92	296	32 (−26 to 91)	.28
Occupational therapy	0.27	62	0.27	70	−9 (−70 to 52)	.78
Plastic surgeon	0.15	70	0.13	28	21 (−28 to 70)	.40
Physiotherapist	0.27	55	0.27	62	−8 (−62 to 46)	.78
Rehabilitation physician	0.05	6	0.06	19	−13 (−39 to 13)	.33
Others	0.20	38	0.25	66	−28 (−103 to 47)	.46
Total outpatient burn care	0.98	778	1.00	768	10 (−243 to 262)	.94
Total costs specialized burn care	1,00	28 154	1,00	26 551	1604 (−2476 to 5684)	.69
*Other health‐care costs*						
Rehabilitation center	0.27	944	0.25	113	831 (−328 to 1989)	.15
Nursing home	0.27	290	0.31	39	251 (0.68 to 503)	.05
General practitioner	0.51	59	0.48	51	8 (−31 to 48)	.68
Home (nursing) care	0.51	1102	0.44	505	597 (−180 to 1374)	.14
Extramural physiotherapy	0.41	196	0.52	358	−162 (−411 to 87)	.20
Others	0.54	286	0.44	205	81 (−118 to 280)	.42
Total other health‐care costs	0.80	2877	0.69	1271	1606 (762 to 2451)	.06
Total health‐care costs	1.00	31 031	1.00	27 821	3210 (−1247 to 7667)	.47
Nonhealth‐care costs						
Work absence (hours) patient	0.59	7721	0.65	8158	−436 (−4074 to 3202)	0.81
Work absence (hours) partner	0.46	2014	0.38	1400	613.44 (−1242.65 to 2469.53)	0.52
Travel costs (km)	1.00	273	1.00	283	−10 (−170 to 149)	0.90
Total nonhealth‐care costs	1.00	10 008	1.00	9841	167 (−3658 to 3991)	0.93
Total societal costs per patient	1.00	41 039	1.00	37 663	3377 (−6229 to 12 982)	0.49

a including reconstructive surgery.

### 4.5 Nonhealth‐care costs and societal costs

The nonhealth‐care costs consisted mainly of loss of economic productivity due to the absence of the patient from work, next to the absence of the partner of the patient from work and travel costs to the burn center (Table 5). The nonhealth‐care costs did not differ significantly between the treatment groups (€10  008 for enzyme alginogel and €9841 for SSD group, *P* = 0.93). Combining the total health care and nonhealth‐care costs resulted in a total mean of societal costs per patient of €41  039 for the enzyme alginogel group and €37  663 for the SSD group (difference: €3377; 95% CI: €6229 to €12 982; *P* = .49). Burn stay costs represented the largest part of the societal costs (48% in the enzyme alginogel group and 51% in the SSD group), followed by nonhealth‐care costs (24% in the enzyme alginogel group and 26% in the SSD group), and treatment costs (11% in the enzyme alginogel group and 10% in the SSD group).

### 4.6 Cost‐utility analysis

**Figure 1:**
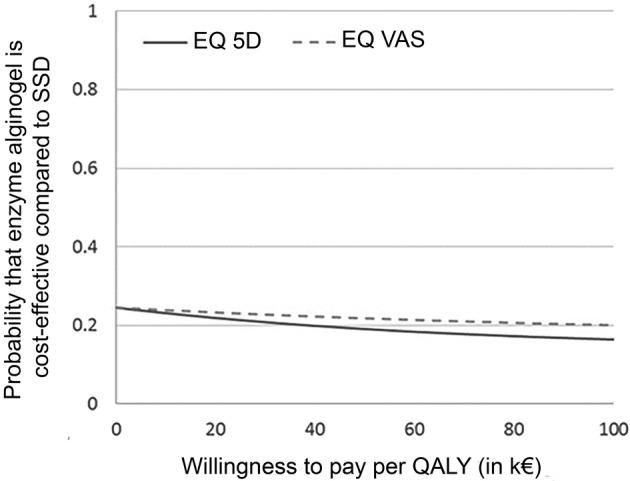
Cost‐effectiveness acceptability curves for enzyme alginogel compared with SSD. QALY, quality‐adjusted life‐year

The combination of nonstatistically higher societal costs and less favorable QALY outcomes after treatment with enzyme alginogel compared with SSD, resulted in a low probability that enzyme alginogel is cost effective compared to SSD. The probability that enzyme alginogel is cost‐effective compared with SSD was less than 25% for all values of the willingness to pay (Figure 1). The same results were obtained when EQ‐VAS utilities were used.

## 5 DISCUSSION

The FLAM study did not show any significant differences in QALYs and health care and societal costs between enzyme alginogel and SSD in the treatment of partial thickness burns over a period of 1 year. Based on the nonsignificant differences in QALYs and costs in favor of SSD, it was concluded that enzyme alginogel is not likely to be cost‐effective compared to SSD (<25%). In both treatment groups, most of the societal costs were caused by burn center stay, absence from work and the treatment. Time to wound healing and need for operation did not differ between the treatment groups, neither for patients with superficial and/or intermediate partial thickness burns nor for patients with deep partial thickness burns as the deepest wound depth.

In the present study, no statistically significant or clinically relevant differences were found between the treatment groups in terms of quality of life when measured with BSHS‐B. Quality of life improved with time for all measured domains. On average, the BSHS‐B scores after burn injury were lowest for the domains “simple abilities,” “heat sensitivity,” and “work” and improved during follow‐up, which is in line with available literature.^27^

In the economic evaluation, we had expected enzyme alginogel to be cost‐effective compared with SSD, because of less dressing changes in the enzyme alginogel group. Although the patients in the enzyme alginogel group did require significantly less dressing changes compared with the SSD group (enzyme alginogel group median of 85% of the days admitted in hospital [range 52‐100%] while in the SSD group almost daily, *P* < .0001).^9^ This difference in dressing changes did not lead to significantly lower costs in the enzyme alginogel group for several reasons. First, wound colonization in the enzyme alginogel group was much more common compared with the SSD group (78% vs 33%, respectively; *P* < .0001), which required daily dressing changes according to our study protocol. For this reason, we think that the a priori assumed advantage of less dressing changes in the enzyme alginogel group was less prominent than expected, as reflected by similar utility scores in both treatment groups. Second, the unit price of enzyme alginogel was higher compared with SSD, which also resulted in comparable total costs of wound care in both treatment groups. Finally, wound care costs in the FLAM study contributed only to a small part of the societal costs (enzyme alginogel 6%, SSD 5.7%; *P* = .42).

In the current study, burn center stay was a major component of the health care and nonhealth‐care costs (societal costs) for both treatment groups, which is in line with other studies on burn care costs.^20^, ^23^, ^28^, ^29^, ^30^ Productivity loss (nonhealth‐care costs) represented the second largest part of societal costs in both treatment groups (enzyme alginogel group 24%, SSD 26%, respectively). Two Dutch studies found comparable results ranging between 25% and 30%.^20^, ^31^ A Spanish study by Sanchez found that loss of productivity accounted for 80% of societal costs.^32^ The higher estimation of costs of productivity loss by Sanchez compared with the FLAM study can partially be explained by a more comprehensive inclusion of nonhealth‐care costs using the human capital approach. In the FLAM study, however, the friction cost method was used, including only actual absenteeism from work in days during a friction period, that is, the time span needed to restore the initial production level, and costs consisted of loss of productivity of the patient and patients' partner, while Sanchez also included loss of productivity of other caregivers. Given the composition of societal costs, future treatment and management of burn wounds should focus on reducing the length of burn center stay and early return to work in order to be cost‐effective, while optimal treatment should be warranted. Developing a wound dressing that does not require daily dressing changes is challenging, because burn wounds might produce considerable amount of wound exudate that require daily (secondary) dressing changes.

Cost studies are important to provide insights on the distribution of costs that, for example, can be used for cost‐reduction measures. Cost‐effectiveness studies on the other hand in which the difference in cost is divided by difference in outcomes between an intervention and its comparator to generate incremental cost‐effectiveness ratio (ICER), provide information on the most favorable balance between cost and health‐care effects.^33^ A systematic review on the economic burden of burn care demonstrated that the majority of the included studies were cost studies and only few studies were cost‐effectiveness studies.^33^ The authors demonstrated that mean total health‐care costs per burn patient in high‐income countries were $88 218 (range $704 to $717 306; median $44024). Noteworthy, the interpretation of these results should be seen in the light of the wide variety of methodological and cost prices that were used in the included studies. The mean total health‐care costs in the current study was lower compared with the above described systematic review, which partially can be explained by the exclusion of %TBSA >30 in the FLAM study. Higher TBSA is associated with higher health‐care costs.^33^

To date, few studies have included health‐care costs in the evaluation of the treatments of partial thickness burns in adult patients. Three RCTs that evaluated different treatments included only cost studies with included cost components that ranged from only material costs to costs including wound treatments, hospital fee, and transportation and pain medications.^34^, ^35^, ^36^ Another RCT on the surgical treatment of partial thickness and full‐thickness burn wounds with dermal substitutes and split skin graft in combination with topical negative pressure performed a cost‐minimization analysis to compare difference in costs. No cost‐effectiveness analyses were performed because there were no significant differences in the studied effect (elasticity).^20^ This study comprehensively assessed the costs including treatments, hospital stay, clinical consultations, other health‐care costs (eg, general practitioner) and absence from work. The authors found no significant differences between total costs per patients for the studied interventions. Two studies performed a cost‐effectiveness analysis in the treatment of partial thickness burns in adult patients. Sheckter et al used a decision model to study the cost‐effectiveness of enclosed silver dressings (Aquacel Ag [ConvaTec, Skillman, NJ] and Mepilex Ag [Molnlycke Health Care, Gothenburg, Sweden]) compared to SSD.^37^ Costs were based on the quantity of the used material, daily home assistance for dressing changes, and outpatient visits. The incremental cost utility ratio, comparing the difference in costs between both treatments and QALYs, was calculated at $40 168/QALY. Assuming a maximum willingness to pay of $50 000/QALY, authors concluded that enclosed silver dressing were cost‐effective. The results of this study, however, should be interpreted with caution because costs were not based on the individual patients but rather on the volume of used materials to treat 20% TBSA burn wound for a period of 3 weeks, including dressing changes at home if needed. Carayanni et al compared moist exposed burn ointment (MEBO) to standard care consisting of povidone plus Bepanthenol cream (Bayer Consumer Care Ltd, Basel, Switzerland).^38^ This study included direct medical costs related to wound treatments and medical visits by physicians and nurses and length of hospital stay. These costs were compared to reduction in hospital days and time of recovery. MEBO was found to result in nonsignificantly lower total costs than standard care and better effectiveness. Overall, it can be concluded that there is a wide variety between studies in regard to which costs and health‐care effects are used in the economic evaluation.

To the best of our knowledge, the FLAM study is the only study that comprehensively studied the clinical effectiveness, quality of life, and cost‐effectiveness of two standard treatments in the treatment of partial thickness burns for a follow‐up period of 1 year. Our study had some limitations. First, the current study was not powered to detect relevant differences in quality of life or costs. Second, data on the daily dressing changes were missing in less than 10% and data on QALYs (EQ‐5D‐5L and EQ‐VAS) were missing in 14%, 17%, and 23% at, respectively, 3, 6, and 12 months postburn. As advocated, however, multiple imputation was used to handle these missing data.^39^ Third, the follow‐up period of this trial was 1 year, which does not cover the long‐term effects of both treatments on quality of life and costs. However, no significant differences were found in quality of life and costs between the treatment groups at 12 months postburn. Since burn scar maturation and recovery is (nearly) completed at that point in patients with partial thickness burns, it is not expected that there are significant differences in quality of life and costs beyond 1 year postburn.

In conclusion, no significant differences were found between enzyme alginogel and SSD in regard to burn‐specific and general quality of life. From a societal perspective, treatment of partial thickness burns with enzyme alginogel is unlikely to be cost‐effective compared with SSD. Finally, from an economic perspective, treatment and management of partial thickness burns should focus on reducing length of hospital stay and early return to work, to achieve optimal outcome.

## CONFLICT OF INTEREST

None of the authors have any potential financial conflicts of interest to disclose.

**Abbreviation**
BSHS‐B: Burn Specific Health Scale‐Brief
HRQoL: Health‐related quality of life
ICER: incremental cost‐effectiveness ratio
SSD: silver sulfadiazine
TBSA: total body surface area
QALYs: quality adjusted life years
VAT: Visual Analogue Thermometer
